# Utilization of Benchtop Next Generation Sequencing Platforms Ion Torrent PGM and MiSeq in Noninvasive Prenatal Testing for Chromosome 21 Trisomy and Testing of Impact of *In Silico* and Physical Size Selection on Its Analytical Performance

**DOI:** 10.1371/journal.pone.0144811

**Published:** 2015-12-15

**Authors:** Gabriel Minarik, Gabriela Repiska, Michaela Hyblova, Emilia Nagyova, Katarina Soltys, Jaroslav Budis, Frantisek Duris, Rastislav Sysak, Maria Gerykova Bujalkova, Barbora Vlkova-Izrael, Orsolya Biro, Balint Nagy, Tomas Szemes

**Affiliations:** 1 Institute of Molecular Biomedicine, Faculty of Medicine, Comenius University in Bratislava, Bratislava, Slovakia; 2 Department of Molecular Biology, Faculty of Natural Sciences, Comenius University in Bratislava, Bratislava, Slovakia; 3 Geneton Ltd., Bratislava, Slovakia; 4 Department of Computer Science, Faculty of Mathematics, Physics and Informatics, Comenius University in Bratislava, Bratislava, Slovakia; 5 1st Department of Gynaecology and Obstetrics, Faculty of Medicine, Comenius University in Bratislava, Bratislava, Slovakia; 6 Department of Clinical Genetics, Medirex Inc., Bratislava, Slovakia; 7 Outpatient Clinic for Medical Genetics, Nemocnicna Inc., Malacky, Slovakia; 8 1st Department of Obstetrics and Gynecology, Semmelweis University, Budapest, Hungary; Tel Aviv University, Israel, ISRAEL

## Abstract

**Objectives:**

The aims of this study were to test the utility of benchtop NGS platforms for NIPT for trisomy 21 using previously published z score calculation methods and to optimize the sample preparation and data analysis with use of *in silico* and physical size selection methods.

**Methods:**

Samples from 130 pregnant women were analyzed by whole genome sequencing on benchtop NGS systems Ion Torrent PGM and MiSeq. The targeted yield of 3 million raw reads on each platform was used for z score calculation. The impact of *in silico* and physical size selection on analytical performance of the test was studied.

**Results:**

Using a z score value of 3 as the cut-off, 98.11% - 100% (104-106/106) specificity and 100% (24/24) sensitivity and 99.06% - 100% (105-106/106) specificity and 100% (24/24) sensitivity were observed for Ion Torrent PGM and MiSeq, respectively. After *in silico* based size selection both platforms reached 100% specificity and sensitivity. Following the physical size selection z scores of tested trisomic samples increased significantly—p = 0.0141 and p = 0.025 for Ion Torrent PGM and MiSeq, respectively.

**Conclusions:**

Noninvasive prenatal testing for chromosome 21 trisomy with the utilization of benchtop NGS systems led to results equivalent to previously published studies performed on high-to-ultrahigh throughput NGS systems. The *in silico* size selection led to higher specificity of the test. Physical size selection performed on isolated DNA led to significant increase in z scores. The observed results could represent a basis for increasing of cost effectiveness of the test and thus help with its penetration worldwide.

## Introduction

The last few years have been marked by rapid progress in the field of non-invasive prenatal diagnostics. Since 1997 when the presence of cell-free fetal DNA (cffDNA) in maternal circulation was discovered [1] different applications based on it progressed to clinical diagnostics, including fetal gender determination [2], fetal RhD status determination [3], and fetal aneuploidy screening [4]. Fetal aneuploidy screening has become widely available with the advance of the next generation sequencing (NGS) technology. In recent studies non-invasive prenatal tests (NIPT) for common trisomies of chromosomes 21, 18 and 13 (T21, T18 and T13) reached almost 100% sensitivity and specificity [5–9]. Currently different tests are offered on a commercial basis and in most cases these are based on whole genome sequencing on ultrahigh throughput NGS platforms. In the majority of laboratories Illumina HiSeq systems are in use for these tests, but other NGS systems achieve similar sensitivity and specificity, including Illumina GIIx [10], SOLiD [11] and Ion Proton [12, 13]. All these NGS systems belong to high to ultrahigh throughput category of NGS systems and are associated with high initial investment into laboratory infrastructure as well as high running and maintenance costs. Moreover, due to their high throughput large numbers of samples are required to reach cost-effectiveness. The lack of validation studies performed on low to middle throughput benchtop NGS systems (Ion Torrent PGM, MiSeq) limits their utilization for NIPT in laboratories with limited budget or low sample turnover. Only recently, studies testing the feasibility of utilization of Ion Torrent PGM, have been published and their results proved the potential of the benchtop NGS sequencers for NIPT, too. However, these studies analyzed only low numbers of euploid:aneuploid samples (4:9 and 4:4, respectively) [14, 15]. Although a z score is widely accepted as the standard parameter used for the detection of trisomic samples, there is no consensus about the selection of a z score calculation method. In the study performed by Jiang et al. four different approaches to z score calculation were compared and the differences in sensitivity and specificity of detection of chromosome 21 trisomic samples were recorded [16]. In our study, three different z score calculation methods were used and compared: 1—the approach using reads mapped to all chromosomes utilized as a reference [17], 2—the approach using reads mapped to chromosome 14 as a reference [18] and 3—the approach using the reads mapped to an optimal combination of chromosomes with the lowest coefficient of variance for reference samples [8, 19]. The performance of NIPT testing based on the z score is influenced by number of factors, with the most important being lengths of the reads and depth of coverage on the technical side and fetal fraction on the biological side [20]. In previously published validation studies read lengths starting from 36 bp and reads counts starting from 2.2x10^6^ of unique reads have been reported as sufficient for accurate and cost-effective aneuploid sample detection [13, 18, 19].

In a recent large international collaborative study low fetal fraction—below 4%—was determined as the main cause for NIPT test failure. Such a low fetal fraction was found to be present in 0.9% (17/1988) of analyzed samples [21]. According to available information from one of the premium NIPT service provider the proportion of samples with fetal fraction below 4% based on analysis of 11225 samples is 4.8% [22].

A significant impact of size selection of maternal circulating DNA on the increase of fetal fraction (up to 4 times) was previously reported when only DNA fragments below 300 bp were collected and used in cffDNA analysis [23, 24]. Moreover, Lo et al. revealed that the fetal to maternal DNA ratio is highest when a cut-off between 143 and 166 bp is used [25]. For virtual enrichment of fetal DNA fraction in genomic data gained from the analysis of maternal circulating DNA *in silico* size selection could be applied, when only reads of a particular length are utilized in z score calculation. Additionally, currently available technologies allow size selection of DNA fragments with high specificity and reproducibility (AmPure XP Reagent beads, LabChip XT system, Pippin Prep system) [26]. Based on the current detailed knowledge about the overrepresentation of fetal DNA in certain size fractions selecting specific size fractions using mentioned technologies could be the means to enrich circulating DNA and thus minimize or even eliminate problems associated with the analysis of samples with low fetal fraction.

The aims of this study were to test the utility of benchtop NGS platforms for NIPT for trisomy 21 using previously published z score calculation methods and to optimize the sample preparation and data analysis with the use of *in silico* and physical size selection methods.

## Materials and Methods

### Ethical committee approval

All parts of the study have been approved according to Slovak legislation as well as international demands for ethical review. The study was approved by the Ethical Committee of the St. Cyril and Method Hospital in Bratislava (decision from 18. October 2012). Patients were included in the study after signing the written informed consent approved by the ethics committee.

### Samples

Peripheral blood samples were taken from 130 pregnant women at 12–23 weeks of pregnancy. Of all tested samples 24 were from pregnant women with previously confirmed chromosome 21 trisomy by invasive methods (amniocentesis, chorionic villi sampling), 50 samples were from pregnant women without elevated risk of fetal chromosome 21 trisomy (risk lower than 1:260) and 56 from pregnant women with high risk of having fetus with chromosome 21 trisomy (1:1.25–1:260).

### Sample processing and DNA extraction

Blood samples were collected in a K3 EDTA Vacutainer (BD, Plymouth, UK) tubes and stored at 4–8°C for up to 24 h before plasma separation. The plasma was separated by two step centrifugation at 2200 g and 16000 g each for 10 min and stored at –20°C until further processing. The cell-free DNA was isolated from 1.8 ml of maternal plasma using a QIAamp Circulating Nucleic Acid Kit (Qiagen, Hilden, Germany). A final elution volume of 85 μl and 56 μl was used for samples analyzed during the validation phase on Ion Torrent PGM and MiSeq, respectively. For physical size selection protocol the DNA was isolated from second plasma aliquot from 10 samples with chromosome 21 trisomy and eluted to 56 μl of molecular biology grade water.

### Physical size selection of DNA samples

Fifty-five μl of Agencourt AMPure XP Reagent beads (Beckman Coulter, Brea, CA, USA) was added to 50 μl of isolated plasma DNA and the solution was mixed thoroughly. The solution was incubated for 5 minutes at room temperature. The tube was placed into a magnetic rack for 5 minutes and the supernatant was transferred into a new tube and 1.8 x volume of beads was added. The solution was mixed by pipetting up and down and incubated for 5 minutes at room temperature. The tube was placed into the magnetic rack for 5 minutes. The pellet was washed with 100 μl 70% ethanol and dried. The pellet was resuspended in 79 μl or 50 μl of Low TE solution and incubated for 5 minutes at room temperature. The tube was placed into the magnetic rack for 5 minutes and supernatant was collected and used in library preparation on Ion Torrent PGM and MiSeq, respectively. Five of the size selected DNA samples were used for Ion Torrent PGM and five for MiSeq library preparation.

### Plasma DNA sequencing using Ion Torrent platform

For library preparation, the Ion Plus Fragment Library Kit (Life Technologies, Carlsbad, CA, USA) was utilized with the original protocol starting from the end repair step (79 μl starting volume). After the end repair step 100 μl of the solution was purified by adding 2.5 x volume of Agencourt AMPure XP Reagent beads. As samples were analyzed individually on either Ion 318 Chip or Ion 318 Chip v2 (Life Technologies) non-barcoded adapters were used in the adapter ligation step. After adapter ligation and nick-repair, 100 μl of the solution was purified in two steps by adding 1.0 x volume and 1.6 x volume of Agencourt AMPure XP Reagent beads. The prepared library was amplified for 15 cycles and purified in two steps by adding 1.6 x and 1.8 x volume of Agencourt AMPure XP Reagent beads. The library was quantified by Ion Library TaqMan Quantitation Kit (Life Technologies) and diluted according to the manufacturer’s recommendations. In emulsion PCR, the DNA library was amplified using Ion PGM Template OT2 200 Kit (Life Technologies). The Ion PGM Sequencing 200 Kit v2 (Life Technologies) with the original protocol was used for sequencing.

### Plasma DNA sequencing using MiSeq platform

The DNA library was prepared according to the TruSeq Nano protocol (Illumina, San Diego, CA, USA) starting from the end repair step (50 μl starting volume). Briefly, 100 μl of the solution after the end repair step was purified with Sample Purification Beads by adding 0.8 x volume and 1.8 x volume of undiluted magnetic beads in two steps. Sample multiplexing was used according to the TruSeq Nano low throughput scheme. After A-tailing and index ligation, the library was purified with Sample Purification Beads twice by adding 1.0 x volume in two separate purification steps. Subsequently, the library was amplified using 8 cycles. The amplified library was purified with Sample Purification Beads by adding 1.0 x volume once. The final libraries were quantified using the Qubit 2.0 Fluorometer (Life Technologies, Carlsbad) and Qubit dsDNA HS assay kit (Invitrogen, Eugene, Oregon, USA) and quality checked on the 2100 Bioanalyzer (Agilent Technologies, Waldbronn, Germany) with use of the High Sensitivity DNA analysis kit (Agilent Technologies, Lithuania). The libraries were normalized to 4 nmol.l^-1^, and 8–10 samples were pooled together and denatured according to the standard protocol. The final library pool was analyzed on MiSeq system using Miseq Reagent kit v3 (Illumina) with pair-end run setting of 2x100 cycles.

### NGS data analysis

Ion Torrent produced fastq files (one per sample) were processed as follows. First, the (remnants of) adapters and poor quality ends from each read were removed using the trimmomatic program [27]. Additionally, this program removed all reads that were shorter than 35 bp after this trimming. More particularly, the following command was used—java -jar /usr/local/tools/trimmomatic-0.32/trimmomatic-0.32.jar SE -threads 1 -phred33 input.fastq output.fastq HEADCROP:11 SLIDINGWINDOW:5:25 MINLEN:35. Moreover, since each read could have been shortened by a different amount (this was determined by the quality of the read's end), original uncut length for each read produced by the above program was stored. This length was later used in the read length based filtering (i.e., reads up to 140 bp, 145 bp, 150 bp, etc.). Subsequently these reads were mapped to the unmasked reference human genome (hg19) using a Bowtie2 algorithm [28]. MiSeq produced fastq files (two per sample) were directly mapped using the bowtie2 algorithm (default parameters); there was no trimming performed in this case. Reads with mapping quality of 40 or higher were retained for further data processing. Next, unique reads were processed to eliminate the GC bias according to Liao et al. 2014 [13] with exclusion of the intrarun normalization. To assess the importance of GC correction, the coefficient of variation (CV) values of chromosome 21, calculated according to Chiu et al. [17] of the training set before and after GC correction were calculated. Because of the different read count in each sample, the CV values were calculated from the fractional genomic representation instead. Before the GC correction, the CV values were 0.93% and 0.83%; after the application of GC correction the CV values were 0.71% and 0.7% for Ion Torrent and MiSeq, respectively. In training set 60 randomly chosen euploid samples were used.

To calculate the z scores, the GC corrected data and three previously published methods for z score calculation were used. The difference between z score calculation methods was in the utilization of different chromosomes or chromosomes sets used as reference for mapping sequencing reads. The first method used all chromosomes (except chromosomes 13, 18 and 21) [17], the second used chromosome 14 [18] and the third used an optimal combination of chromosomes with the lowest coefficient of variance for reference samples [8, 19]. Note that in the third case the selected chromosomes for internal reference were 7, 11, 20, 22 and 7, 11, 17, 20 for Ion Torrent PGM and MiSeq, respectively. The application of the first and second z score calculation method is well described in their referred publications. The application of third method is described below. The matrix of chromosome counts of 60 training euploid samples (each row corresponds with one sample and the columns contain chromosomes in the order 1:22, X, Y) was used to determine the best internal reference chromosomes (IR). A candidate IR was any combination of chromosomes excluding combinations containing chromosomes 13, 18, 21, X and Y (thus, there are 2^19 candidate IRs). For each candidate IR its CV value was calculated as follows. First, for each training sample the ratio between the read count of chromosome 21 and the summed read count of candidate IR chromosomes was calculated (thus 60 values as there are 60 training samples were obtained). Then the mean M and standard deviation S of these 60 values were calculated. The CV value of the candidate IR was given by S/M. It was done for all candidate IRs, and the one with the lowest CV value was chosen. Intuitively, this was the IR under which the euploid training samples appear to be most compact. Using the IR the ratio between the read count of chromosome 21 and the summed read count of IR chromosomes for each test sample was calculated, and the values M and S (associated with the chosen IR) were chosen to obtain the z-score of any test sample in the usual manner.

Fetal fraction was calculated in trisomic samples according to the method of Rava et al. [29]. To test the effect of *in silico* size selection, we filtered out reads of a size greater than 140 bp and then in 5 bp steps up to 180 bp, and used the remaining reads for z score calculation.

### Statistical analysis

Data were analyzed using Microsoft Excel 2007^®^ (Microsoft, Redmont, WA, USA), OriginLab9 (OriginLab, Northampton, MA, USA) and GraphPad Prism^®^ version 6 (GraphPad Software, La Jola, CA, USA). Results of z score calculations with three tested methods were compared using ANOVA (euploid samples) and the Friedman test (trisomic samples) and results of utilization of original and size selected data or samples were compared using a paired t-test. The p value < 0.05 was considered as significant. The basic data used in statistical analyses are summarized in S1 and S2 Tables.

## Results

### Sequencing data collection and primary analysis

On average 5.829 ± 1.026 million and 3.098 ± 0.951 million raw reads were obtained from sequencing, with average read lengths of 179 ± 7 bp and 172 ± 7 bp on Ion Torrent PGM and MiSeq, respectively. To reach similar conditions for comparison of the two tested benchtop NGS platforms in the z score calculation step the gained raw read counts were normalized and a maximum of 3 million of reads were randomly selected in all samples. After filtering steps, the counts of final reads used in z score calculations decreased from 3 million per sample to an average of 2.160 ± 0.065 million and 2.099 ± 0.221 million for Ion Torrent PGM and MiSeq, respectively.

### Validation of utilization of Ion Torrent PGM and MiSeq

After z score calculations with the three methods, the specificity of the test varied between 98.11%–100% (95% CI—93.35% to 100%) and 99.06%–100% (95% CI: 94.86% to 100%) and sensitivity of the test was 100% and 100% (95% CI: 85.75% to 100.00%) for Ion Torrent PGM and MiSeq, respectively (Table 1).

**Table 1:** The performance of three z score calculation methods for noninvasive detection of chromosome 21 trisomy.

z score calculation method	1*		2*		3*	
	Sensitivity	Specificity	Sensitivity	Specificity	Sensitivity	Specificity
Ion Torrent PGM all reads	100% (24/24)	99.06% (105/106)	100% (24/24)	100% (106/106)	100% (24/24)	98.11% (104/106)
Ion Torrent PGM up to 160 bp reads	100% (24/24)	100% (106/106)	100% (24/24)	100% (106/106)	100% (24/24)	100% (106/106)
MiSeq all reads	100% (24/24)	100% (106/106)	100% (24/24)	99.06% (105/106)	100% (24/24)	100% (106/106)
MiSeq up to 155 bp reads	100% (24/24)	100% (106/106)	100% (24/24)	100% (106/106)	100% (24/24)	100% (106/106)

*—1—the approach using reads mapped to all chromosomes utilized as a reference [17], 2—the approach using reads mapped to chromosome 14 as a reference [18] and 3—the approach using the reads mapped to optimal combination of chromosomes with the lowest coefficient of variance for reference samples [8, 19].

**Fig 1:**
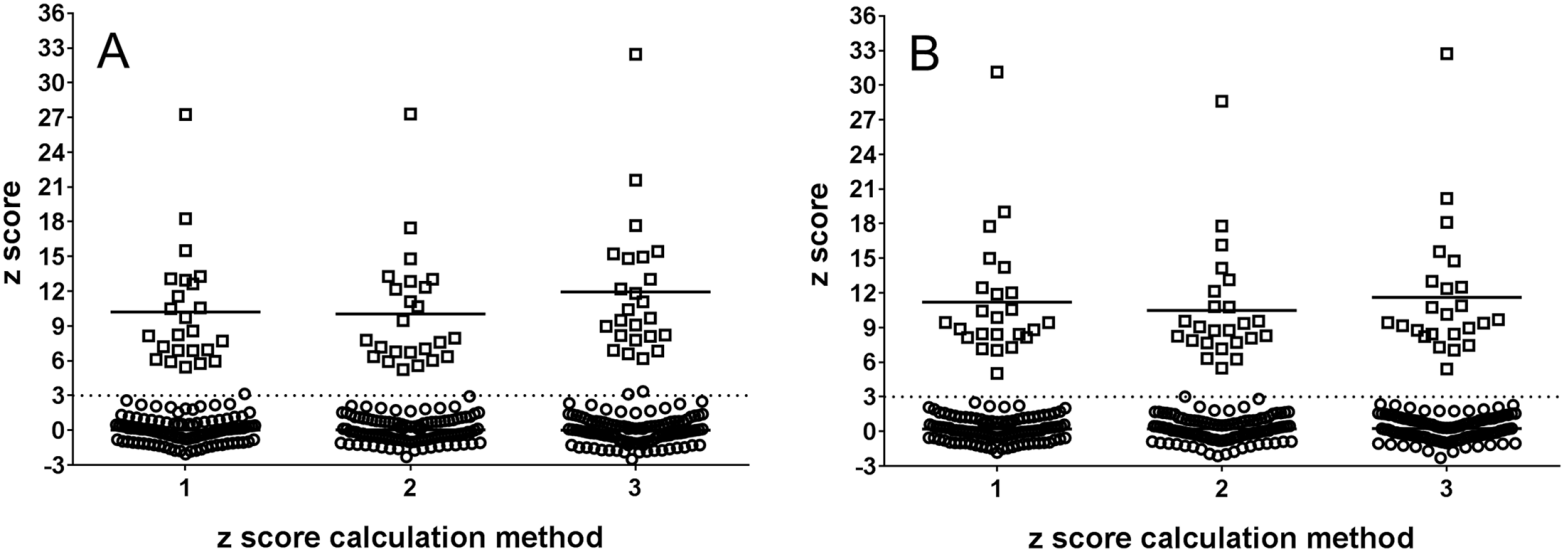
Z score values of samples calculated by three different previously published methods. **□** –trisomic samples, **○** –euploid samples, horizontal line—mean z score value of trisomic samples. Dotted lines represent the standard limit for identification of a trisomic sample (z score = 3). A—Ion Torrent PGM analyzed samples, B—MiSeq analyzed samples.

When z score values of trisomic samples calculated by the three chosen z score calculation methods were compared, the difference was found to be statistically significant (p < 0.0001, for both Ion Torrent PGM and MiSeq analyses) while no significant difference was recorded when comparing z scores of euploid samples. According to the results of a post hoc Dunn´s multiple comparison test and the calculation of the difference of average z scores of trisomic and euploid samples the method 3 achieved the highest difference between z scores of trisomic and euploid samples (Fig 1) and was used in the next steps in the study.

### *In silico* size selection based optimization of data analysis and test performance

**Fig 2:**
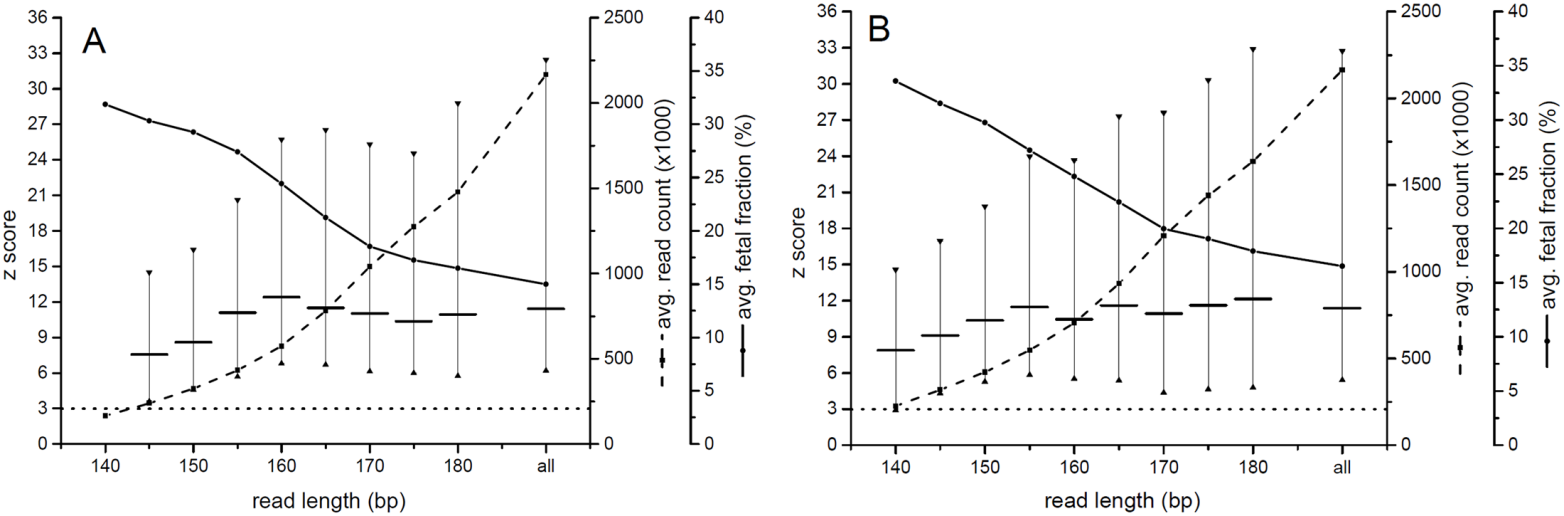
Determination of optimal *in silico* size selection limit for sequencing reads to be used in z score calculation. Dotted lines represent the standard limit for identification of a trisomic sample (z score = 3). A—Ion Torrent PGM analyzed samples, B—MiSeq analyzed samples.

Limiting read lengths led to a substantial drop in read counts; nevertheless, z scores reached the highest average values when the reads up to 160 bp and 155 bp were used in z score calculations for Ion Torrent PGM and MiSeq, respectively (Fig 2).

**Fig 3:**
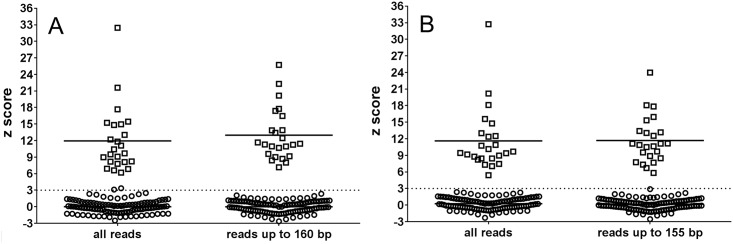
Z score values of trisomic and euploid samples before and after *in silico* size selection of sequencing reads. **□** –trisomic samples, **○** –euploid samples, horizontal line—mean z score value). Dotted lines represent the standard limit for identification of a trisomic sample (z score = 3). A—Ion Torrent PGM analyzed samples, B—MiSeq analyzed samples.

When using only reads of up to 160 bp for Ion Torrent PGM and 155 bp for MiSeq for z score calculation the specificity as well as sensitivity of trisomy 21 detection of both platforms reached 100% because a decrease of z score values of all false positive samples was observed. On the other hand z scores of trisomic samples increased significantly (p = 0.0407) on Ion Torrent PGM and were not significantly different on MiSeq despite the fact that the number of reads used for z score calculation dropped to approximately 1/4 in comparison to numbers of reads used for z score calculation before *in silico* size selection (26.36% and 24.31% on Ion Torrent PGM and MiSeq, respectively; Fig 3).

### Fetal fraction determination

The fetal fractions calculated in samples with confirmed chromosome 21 trisomy were 8.12%–42.62% (median = 13.19%) and 7.85%–47.49% (median = 13.86%). After *in silico* size selection, which resulted in the change of only so-called “effective” fetal fraction while the fetal fraction physically present in the analyzed sample remain unchanged, the effective fetal fractions increased to 14.08%–50.66% (median = 22.35%) and 13.82%–56.89% (median = 26.09%) for Ion Torrent PGM and MiSeq analyzed samples, respectively. The increase of the effective fetal fraction after *in silico* size selection was statistically significant in samples analyzed on both benchtop NGS platforms (p < 0.0001).

### Physical size selection based optimization of sample processing and test performance

**Fig 4:**
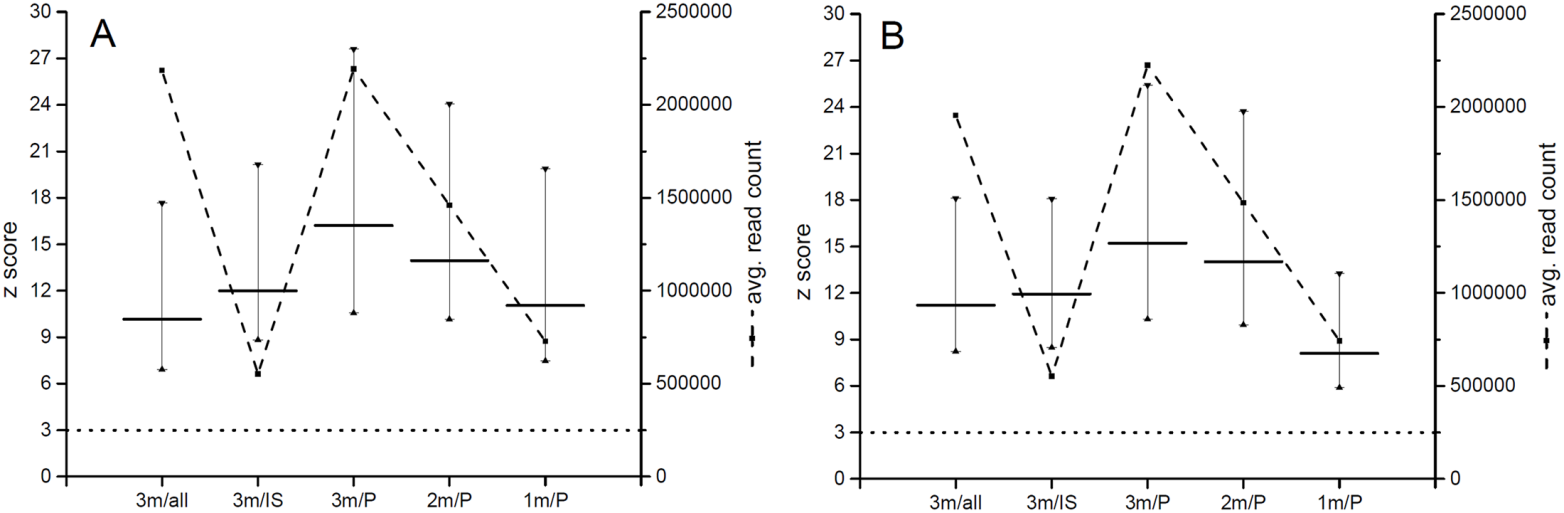
Z score values calculated from reads without size selection (all), after *in silico* size selection (IS) and after physical size selection (P). Horizontal lines represent mean z score value calculated from 3, 2 and 1 million raw reads (3m, 2m, 1m). Dotted lines represent the standard limit for identification of a trisomic sample (z score = 3). A—Ion Torrent PGM analyzed samples, B—MiSeq analyzed samples.

The z scores calculated for samples without physical size selection using 3 million raw reads and after physical size selection using 3 million and 2 million raw reads were found to be significantly different (p < 0.05), with significantly higher z scores found in physically size selected samples. Statistically not significantly different z scores were obtained when 3 million raw reads without and 1 million raw reads with physical size selection of identical samples were compared (Fig 4).

## Discussion

To our knowledge this is the first study focused on the validation of benchtop NGS systems utilization in NIPT. Previously only two feasibility studies were performed on Ion Torrent PGM. These studies analyzed small scale sample groups (< 10 samples per euploid/aneuploid groups) [14, 15] and therefore their results cannot be directly compared to previously published large scale validation studies [5–7, 11, 13, 16, 21]. In contrast, we have analyzed more than 100 samples from low risk and high risk euploid pregnancies and over 20 samples from pregnancies with previously confirmed chromosome 21 trisomy. For the detection of trisomic samples three selected methods based on z score calculation were used as the accepted standard in NIPT for most common aneuploidies. Our results showed that two benchtop NGS platforms, Ion Torrent PGM and MiSeq, could be safely used for NIPT reaching similarly high sensitivity and specificity as high and ultrahigh throughput NGS systems [5–7, 11, 13, 16, 21]. Nevertheless, our data raise several questions that need to be addressed before the utilization of benchtop NGS platforms in NIPT with appropriate confidence. One of the questions is what coverage is necessary for the correct identification of an extra chromosome 21 (as well as 18 and 13). In our study up to 3 million raw reads per sample were targeted by design. In reality in some samples analyzed on both platforms this could not be reached and therefore on average 2.996 million (SD = ± 0.037) and 2.793 million (SD = ± 0.280) raw reads were used in filtering steps and subsequent z score calculation on Ion Torrent PGM and MiSeq, respectively. With this limited number of reads the sensitivity was 100% and specificity of the test was close to 100% when standard filtering criteria were used for read mapping, quality filtering and GC-bias reduction before z score calculation. Using three different z score calculation methods, 0 to 2 and 0 to 1 false positive samples were identified among euploid samples analyzed on Ion Torrent PGM and MiSeq, respectively.

According to the previously published data there is a difference in the abundance of differently sized DNA in between mother and fetus originating DNA [4, 25]. Additionally, it was shown that size selection of specific DNA fragment size could be used to significantly increase the fetal fraction [23, 24]. It is widely accepted that low fetal fraction is the leading cause for false negative results in the NIPT test or the test failure [21]. To test whether *in silico* size selection could lead to an increase of the fetal fraction and subsequently to higher specificity and sensitivity of the test size selection of sequencing reads from 140 bp up to 180 bp in step of 5 bp was performed. This analysis led to the highest z scores of trisomic samples when cut off values of 160 bp and 155 bp were used for Ion Torrent PGM and MiSeq reads, respectively (Fig 2). The difference between Ion Torrent PGM and MiSeq cut off values mimics the difference of average read lengths that was recorded on raw sequencing reads—179 bp and 172 bp, respectively. After *in silico* size selection and z score calculation based on reads up to 160 bp and 155 bp was performed on the whole set of samples (euploid and trisomic) no false positives were identified and both sensitivity and specificity of the test reached 100%. Moreover, although only approximately 25% of original filtered reads were used in z score calculation after *in silico* size selection z scores of trisomic samples were significantly higher (p < 0.05) or not significantly different in the case of Ion Torrent PGM and MiSeq data, respectively. This finding shows that it is possible to decrease the number of reads needed for NIPT for chromosome 21 trisomy by a factor of 4 without decreasing the power of the test if a specific size fractionation of cffDNA could be performed as part of the sample preparation procedure. Therefore in our study we decided to introduce physical size selection into the sample processing procedure also. For size selection the AMPure beads, which are routinely used in size selection steps of NGS library preparation protocols, were utilized. We tested several concentrations of AMPure beads and optimal results with dominant enrichment of DNA fragment sizes around 155–160 bp were identified when a two step protocol was used. In all the cases, a significant increase of z score values was detected when the z scores calculated from 3 million raw reads of samples without physical size selection were compared to z scores calculated from 3 million and 2 million raw reads of samples after physical size selection. Z score values were found to be not significantly different when z scores calculated from 3 million raw reads of samples without physical size selection were compared to z scores calculated from 1 million raw reads of samples after physical size selection. This finding was consistent between samples analyzed on Ion Torrent PGM as well as MiSeq. Therefore, with such a simple two-step size selection the number of reads needed for NIPT for chromosome 21 trisomy could be decreased by a factor of 3 without a decrease in test power. In the end both *in silico* and physical size selection methods bring similar results, but each of the performed size selection procedures has its pros and cons. Implementation of *in silico* size selection into NIPT performing laboratory routine is associated with simple adding of a filtering step focused on sequencing read length into the data analysis pipeline without changing the validated sample preparation protocol. Moreover, as no additional manipulation with a sample is needed, the risk of sample contamination during its processing is minimized. On the other hand, inclusion of the physical size selection in NGS library preparation as a routinely performed step with consumables and chemistry that are normally used in NGS allows simultaneous analysis of increased number of samples per run. That leads to the increased cost efficiency of NIPT without compromizing sensitivity and specificity of the test.

To sum up, currently there are only few laboratories over the world that perform NIPT in their laboratories and this testing is performed almost exclusively on high-to-ultrahigh throughput NGS systems. The results of our study showed that with future larger scale validation studies and further sample preparation procedure tuning low-to-middle throughput (benchtop) NGS systems could be utilized with similar cost efficiency and sensitivity and specificity for routine NIPT and help with better global accessibility of the test.

## Supporting Information

S1 Table Data used in basic statistics and evaluation of detection of trisomic samples analyzed on Ion Torrent PGM system. (XLSX)

S2 Table Data used in basic statistics and evaluation of detection of trisomic samples analyzed on MiSeq system. (XLSX)

## References

[1] Lo YM, Corbetta N, Chamberlain PF, Rai V, Sargent IL, Redman CW, et al Presence of fetal DNA in maternal plasma and serum. Lancet. 1997;350: 485–7. 9274585 10.1016/S0140-6736(97)02174-0

[2] Lo YM, Tein MS, Lau TK, Haines CJ, Leung TN, Poon PM, et al Quantitative analysis of fetal DNA in maternal plasma and serum: implications for noninvasive prenatal diagnosis. Am J Hum Genet. 1998;62: 768–75. 9529358 10.1086/301800 PMC1377040

[3] Lo YM, Hjelm NM, Fidler C, Sargent IL, Murphy MF, Chamberlain PF, et al Prenatal diagnosis of fetal RhD status by molecular analysis of maternal plasma. N Engl J Med. 1998;339: 1734–8. 9845707 10.1056/NEJM199812103392402

[4] Fan HC, Blumenfeld YJ, Chitkara U, Hudgins L, Quake SR. Noninvasive diagnosis of fetal aneuploidy by shotgun sequencing DNA from maternal blood. Proc Natl Acad Sci U S A. 2008;105: 16266–71. 10.1073/pnas.0808319105 18838674 PMC2562413

[5] Palomaki GE, Kloza EM, Lambert-Messerlian GM, Haddow JE, Neveux LM, Ehrich M, et al DNA sequencing of maternal plasma to detect Down syndrome: an international clinical validation study. Genet Med. 2011;13: 913–20. 22005709 10.1097/GIM.0b013e3182368a0e

[6] Nicolaides KH, Syngelaki A, Ashoor G, Birdir C, Touzet G. Noninvasive prenatal testing for fetal trisomies in a routinely screened first-trimester population. Am J Obstet Gynecol. 2012;207: 374e1–6. 23107079 10.1016/j.ajog.2012.08.033

[7] Ehrich M, Deciu C, Zwiefelhofer T, Tynan JA, Cagasan L, Tim R, et al Noninvasive detection of fetal trisomy 21 by sequencing of DNA in maternal blood: a study in a clinical setting. Am J Obstet Gynecol. 2011;204: 205e1–11. 21310373 10.1016/j.ajog.2010.12.060

[8] Bianchi DW, Platt LD, Goldberg JD, Abuhamad AZ, Sehnert AJ, Rava RP. Genome-wide fetal aneuploidy detection by maternal plasma DNA sequencing. Obstet Gynecol. 2012;119: 890–901. 10.1097/AOG.0b013e31824fb482 22362253

[9] Ashoor G, Syngelaki A, Wagner M, Birdir C, Nicolaides KH. Chromosome-selective sequencing of maternal plasma cell-free DNA for first-trimester detection of trisomy 21 and trisomy 18. Am J Obstet Gynecol. 2012;206: 322e1–5. 22464073 10.1016/j.ajog.2012.01.029

[10] Stumm M, Entezami M, Trunk N, Beck M, Locherbach J, Wegner RD, et al Noninvasive prenatal detection of chromosomal aneuploidies using different next generation sequencing strategies and algorithms. Prenat Diagn. 2012;32: 569–77. 10.1002/pd.3862 22573401

[11] Faas BH, de Ligt J, Janssen I, Eggink AJ, Wijnberger LD, van Vugt JM, et al Non-invasive prenatal diagnosis of fetal aneuploidies using massively parallel sequencing-by-ligation and evidence that cell-free fetal DNA in the maternal plasma originates from cytotrophoblastic cells. Expert Opin Biol Ther. 2012;12 Suppl 1: S19–26. 10.1517/14712598.2012.670632 22500971

[12] Jeon YJ, Zhou Y, Li Y, Guo Q, Chen J, Quan S, et al The feasibility study of non-invasive fetal trisomy 18 and 21 detection with semiconductor sequencing platform. PLoS One. 2014;9: e110240 10.1371/journal.pone.0110240 25329639 PMC4203771

[13] Liao C, Yin AH, Peng CF, Fu F, Yang JX, Li R, et al Noninvasive prenatal diagnosis of common aneuploidies by semiconductor sequencing. Proc Natl Acad Sci U S A. 2014;111: 7415–20. 10.1073/pnas.1321997111 24799683 PMC4034209

[14] Wang Y, Wen Z, Shen J, Cheng W, Li J, Qin X, et al Comparison of the performance of Ion Torrent chips in noninvasive prenatal trisomy detection. J Hum Genet. 2014;59: 393–6. 10.1038/jhg.2014.40 24919645

[15] Yuan Y, Jiang F, Hua S, Du B, Hao Y, Ye L, et al Feasibility study of semiconductor sequencing for noninvasive prenatal detection of fetal aneuploidy. Clin Chem. 2013;59: 846–9. 10.1373/clinchem.2012.196725 23364181

[16] Jiang F, Ren J, Chen F, Zhou Y, Xie J, Dan S, et al Noninvasive Fetal Trisomy (NIFTY) test: an advanced noninvasive prenatal diagnosis methodology for fetal autosomal and sex chromosomal aneuploidies. BMC Med Genomics. 2012;5: 57 10.1186/1755-8794-5-57 23198897 PMC3544640

[17] Chiu RW, Akolekar R, Zheng YW, Leung TY, Sun H, Chan KC, et al Non-invasive prenatal assessment of trisomy 21 by multiplexed maternal plasma DNA sequencing: large scale validity study. BMJ. 2011;342: c7401 10.1136/bmj.c7401 21224326 PMC3019239

[18] Lau TK, Chen F, Pan X, Pooh RK, Jiang F, Li Y, et al Noninvasive prenatal diagnosis of common fetal chromosomal aneuploidies by maternal plasma DNA sequencing. J Matern Fetal Neonatal Med. 2012;25: 1370–4. 10.3109/14767058.2011.635730 22070770

[19] Sehnert AJ, Rhees B, Comstock D, de Feo E, Heilek G, Burke J, et al Optimal detection of fetal chromosomal abnormalities by massively parallel DNA sequencing of cell-free fetal DNA from maternal blood. Clin Chem. 2011;57: 1042–9. 10.1373/clinchem.2011.165910 21519036

[20] Lo KK, Boustred C, Chitty LS, Plagnol V. RAPIDR: an analysis package for non-invasive prenatal testing of aneuploidy. Bioinformatics. 2014;30: 2965–7. 10.1093/bioinformatics/btu419 24990604 PMC4184262

[21] Palomaki GE, Deciu C, Kloza EM, Lambert-Messerlian GM, Haddow JE, Neveux LM, et al DNA sequencing of maternal plasma reliably identifies trisomy 18 and trisomy 13 as well as Down syndrome: an international collaborative study. Genet Med. 2012;14: 296–305. 10.1038/gim.2011.73 22281937 PMC3938175

[22] Available from: http://www.panoramateszt.hu/panoramatest/szakembereknek.

[23] Hromadnikova I, Zejskova L, Doucha J, Codl D. Quantification of fetal and total circulatory DNA in maternal plasma samples before and after size fractionation by agarose gel electrophoresis. DNA Cell Biol. 2006;25: 635–40. 17132094 10.1089/dna.2006.25.635

[24] Li Y, Zimmermann B, Rusterholz C, Kang A, Holzgreve W, Hahn S. Size separation of circulatory DNA in maternal plasma permits ready detection of fetal DNA polymorphisms. Clin Chem. 2004;50: 1002–11. 15073090 10.1373/clinchem.2003.029835

[25] Lo YM, Chan KC, Sun H, Chen EZ, Jiang P, Lun FM, et al Maternal plasma DNA sequencing reveals the genome-wide genetic and mutational profile of the fetus. Sci Transl Med. 2010;2: 61ra91 10.1126/scitranslmed.3001720 21148127

[26] Quail MA, Gu Y, Swerdlow H, Mayho M. Evaluation and optimisation of preparative semi-automated electrophoresis systems for Illumina library preparation. Electrophoresis. 2012;33: 3521–8. 10.1002/elps.201200128 23147856

[27] Bolger AM, Lohse M, Usadel B. Trimmomatic: a flexible trimmer for Illumina sequence data. Bioinformatics. 2014;30: 2114–20. 10.1093/bioinformatics/btu170 24695404 PMC4103590

[28] Langmead B, Salzberg SL. Fast gapped-read alignment with Bowtie 2. Nat Methods. 2012;9: 357–9. 10.1038/nmeth.1923 22388286 PMC3322381

[29] Rava RP, Srinivasan A, Sehnert AJ, Bianchi DW. Circulating fetal cell-free DNA fractions differ in autosomal aneuploidies and monosomy X. Clin Chem. 2014;60: 243–50. 10.1373/clinchem.2013.207951 24046201

